# Supplementary figures and images for: T-Toxin Virulence Genes: Unconnected Dots in a Sea of Repeats
Source: mBio. 2023 Mar 8;14(2):e00261-23. doi: 10.1128/mbio.00261-23 (PMC10128009; doi:10.1128/mbio.00261-23)

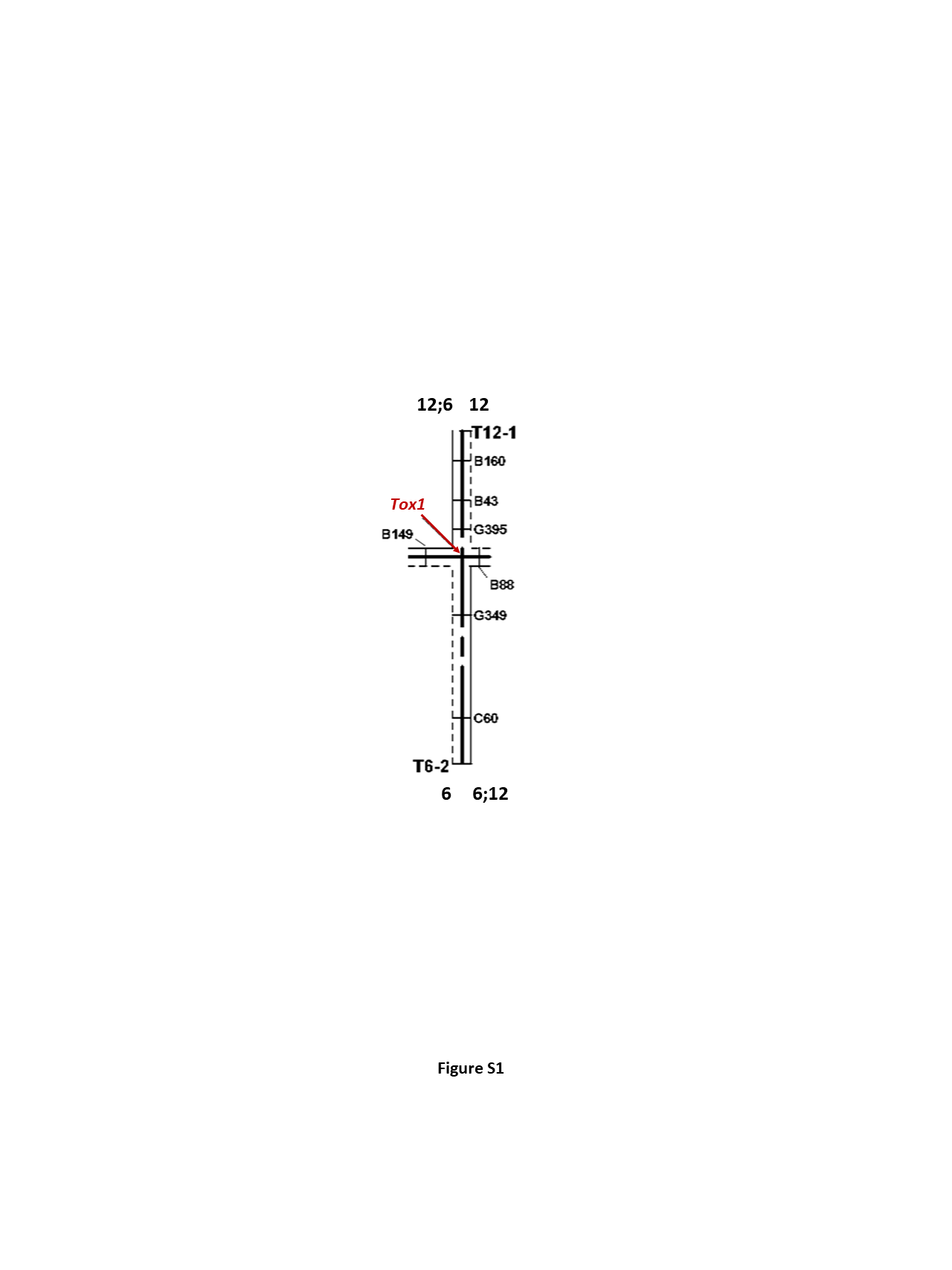

Supplement: Figure S1 [file mbio.00261-23-s0005.tif]

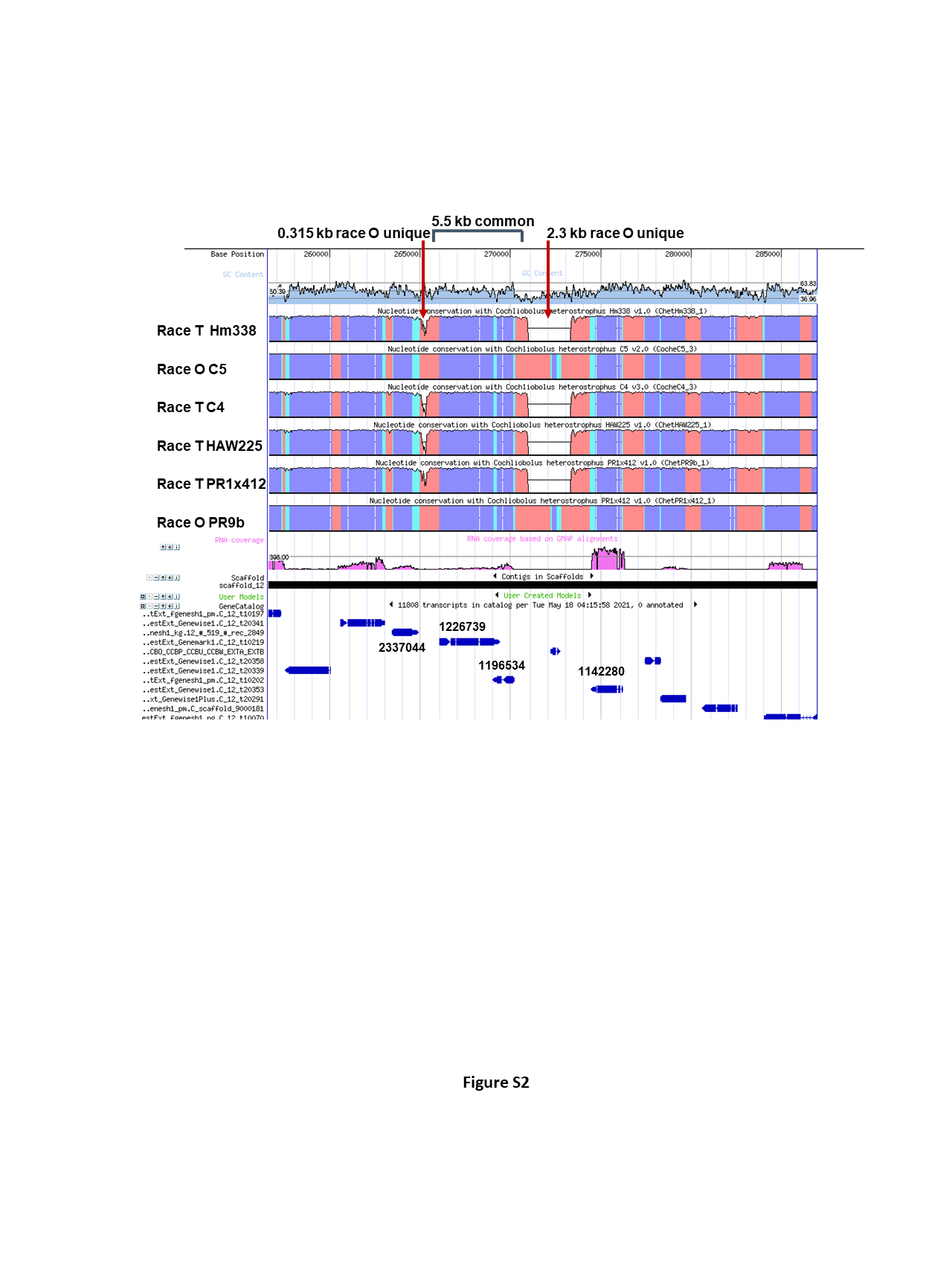

Supplement: FIG S2 [file mbio.00261-23-s0006.tif]

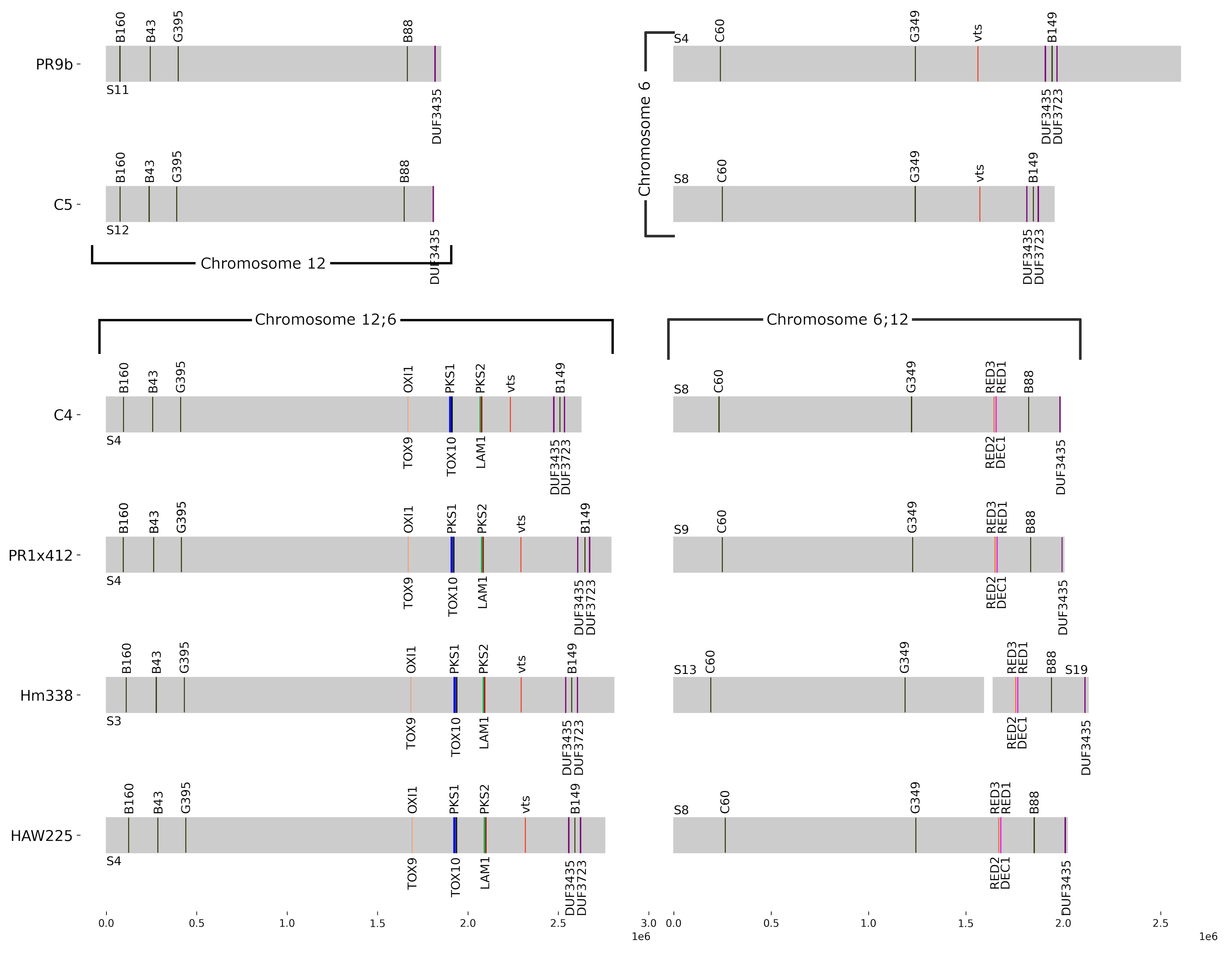

Supplement: FIG S3 [file mbio.00261-23-s0007.tif]
